# The CHA_2_DS_2_-VASc score for predicting atrial fibrillation in patients presenting with ST elevation myocardial infarction: prospective observational study

**DOI:** 10.1590/1516-3180.2018.0431140319

**Published:** 2019-07-22

**Authors:** Fatih Aksoy, Hasan Aydin Baş, Ali Bağcı, Tulay Oskay

**Affiliations:** I MD. Associate Professor, Department of Cardiology, Süleyman Demirel Üniversitesi Tıp Fakültesi, Isparta, Turkey.; II MD. Physician, Department of Cardiology, Isparta Şehir Hastanesi, Isparta, Turkey.; III MD. Physician, Department of Cardiology, Isparta Şehir Hastanesi, Isparta, Turkey.; IV MD. Physician, Department of Cardiology, Merzifon Devlet Hastanesi, Amasya, Turkey.

**Keywords:** Risk factors, Atrial fibrillation, Myocardial infarction

## Abstract

**BACKGROUND:**

Atrial fibrillation (AF) is the most common form of supraventricular arrhythmia following ST-elevation myocardial infarction (STEMI). The CHA_2_DS_2_-VASc and CHADS_2_ scores are used to estimate thromboembolic risk in cases of AF. Their usefulness in predicting the development of AF in patients presenting STEMI is unknown.

**OBJECTIVE:**

To evaluate the predictive value of the CHADS_2_ and CHA_2_DS_2_-VASc scores in patients with AF following STEMI.

**DESIGN AND SETTING:**

This prospective cohort study on 696 patients with STEMI was conducted at a tertiary-level cardiology clinic in a public university hospital.

**METHODS:**

Models including clinical and laboratory parameters were constructed to test the predictive value of CHADS_2_ and CHA_2_DS_2_-VASc scores. Patients were divided into two groups: with and without AF. Predictors of AF were determined using multivariate regression analysis.

**RESULTS:**

In the patients with AF, CHADS_2_ and CHA_2_DS_2_-VASc scores were significantly higher than in those without AF (for both P < 0.001). Factors associated with AF in multivariate analyses included CHA_2_DS_2_-VASc score (odds ratio, OR: 1.48; 95% confidence interval, CI: 1.25-1.75; P < 0.001), peak creatine kinase-myocardial binding (OR: 1.002; 95% CI: 1.00-1.003; P = 0.0024), duration of the coronary intensive care unit stay (OR: 1.69; 95% CI: 1.24-12.30; P = 0.001) and no use of renin-angiotensin system blockers (OR: 2.16; 95% CI: 1.14-4.10; P = 0.0017). Receiver operating characteristic curve analyses showed that CHA_2_DS_2_-VASc scores were signiﬁcant predictors for new-onset AF (C-statistic: 0.698; 95% CI: 0.631-0.765; P < 0.001).

**CONCLUSION:**

CHADS_2_ and CHA_2_DS_2_-VASc scores predicted new AF in patients presenting STEMI.

## INTRODUCTION

Atrial fibrillation (AF) presents increasing prevalence with increasing age and is the most common type of arrhythmia in clinical practice, affecting 1%-2% of the general population.^1^^,^^2^ Thromboembolic events, which can cause death, disability and impaired quality of life, are important complications of AF.^3^ AF is the most common type of supraventricular arrhythmia following ST-segment elevation myocardial infarction (STEMI), and its prevalence is even higher among elderly patients with heart failure and severe left ventricular impairment.^3^ Patients who develop AF following STEMI are at higher risk of stroke and death than are those who do not develop AF. Older age, female gender, low blood pressure, higher heart rate, higher Killip class, history of hypertension, prior myocardial infarction, diabetes mellitus and low ejection fraction can be predisposing factors for the development of AF following STEMI.^3^

The CHA_2_DS_2_-VASc risk score is a cheap and easy-to-use scoring system that is calculated by assigning one point for each of the following: congestive heart failure (ejection fraction < 40%), hypertension, age between 65 and 74 years, diabetes mellitus, vascular disease (myocardial infarction or peripheral arterial disease) and female sex; and two points for a history of stroke or transient ischemic attack (TIA) and age > 75 years. Additionally, the CHA_2_DS_2_-VASc risk score is used to predict the risk of thromboembolism among non-valvular AF patients.^3^

## OBJECTIVE

In this study, we aimed to evaluate the association between the CHADS_2_ and CHA_2_DS_2_-VASc risk scores and the development of AF in patients presenting with STEMI.

## METHODS

In this prospective study, 724 consecutive patients with STEMI who were admitted to the cardiology clinic of Süleyman Demirel University Hospital (a tertiary-level cardiology clinic in Isparta, Turkey) were screened between January 2014 and December 2015. The inclusion criteria included age greater than 18 years and presence of acute STEMI. The exclusion criteria included unstable angina pectoris, non-ST-elevation myocardial infarction, hyperthyroidism, history of AF (paroxysmal, persistent or permanent), moderate to severe heart valve disease, advanced chronic obstructive pulmonary disease, infection, sepsis, rheumatic or inflammatory disease, history of malignancy and use of antiarrhythmic drugs.

**Figure 1.**
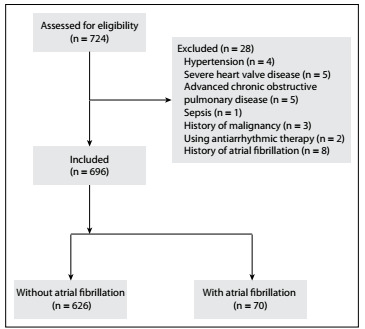
Flow diagram for patient selection.

Out of 724 consecutive patients with acute STEMI, the following were excluded: four patients with hyperthyroidism, five patients with severe heart valve disease, five patients with advanced chronic obstructive pulmonary disease, one patient with sepsis, three patients with a history of malignancy, two patients using antiarrhythmic therapy and eight patients with a history of AF. Therefore, the study cohort consisted of 696 patients with STEMI (Figure 1).

Our institutional ethics committee approved the study (date: July 29, 2011; session number: 25; decision no: 18) and all participants provided written informed consent.

Diagnoses were recorded by the participating physicians based on clinical, electrocardiographic and biochemical (elevated troponin level) criteria. The type of myocardial infarction (ST-elevation versus non-ST-elevation) and situation of unstable angina were homogeneously defined and based on current guidelines.^4^ The CHADS_2_ and CHA_2_DS_2_-VASc risk scores were calculated according to current guidelines.^3^

Each patient was questioned about major cardiovascular risk factors, including family history of coronary artery disease, current smoking status, hyperlipidemia, hypertension, diabetes mellitus and obesity. A family history of coronary artery disease was defined as manifestation of the disease in first-degree male relatives younger than 55 years of age or in first-degree female relatives younger than 65 years. Hyperlipidemia was defined as fasting total cholesterol level > 200 mg/dl or pharmacotherapy with lipid-lowering agents. Hypertension was defined as systolic blood pressure ≥ 140 mmHg and/or diastolic blood pressure ≥ 90 mmHg, measured before hospitalization or pharmacotherapy with antihypertensive drugs. Diabetes mellitus was defined as fasting plasma glucose ≥ 126 mg/dl or pharmacotherapy with insulin or oral antidiabetic agents. Obesity was defined as body mass index > 30 kg/m^2^. Patients who were smoking prior to hospitalization were deemed to be smokers.

Clinical data on the patients, their previous medication histories and medications started after hospitalization were recorded. The patients were divided into two groups: those with AF and those without AF. A 12-lead electrocardiogram was recorded upon admission to the hospital. AF was deﬁned as an irregular rhythm with the absence of discrete P waves in the 12-lead electrocardiogram.^3^ Patients were followed up through continuous electrocardiography (ECG) monitoring during their stay at the coronary intensive care (CICU), to detect any occurrence of AF, which was defined as an irregular narrow complex rhythm (in the absence of bundle branch block) with absence of discrete P waves. The patients did not undergo continuous ECG monitoring during their stay in the wards, and therefore rhythm follow-up was not evaluated in the wards. An AF episode lasting > 30 seconds during hospitalization at the CICU was taken to be an endpoint.

All patients were treated in accordance with the currently available guidelines.^5^ Primary percutaneous coronary intervention (PCI) was performed on all patients. The patients underwent transthoracic echocardiography, and the left ventricular ejection fraction was calculated by means of Simpson’s method.^6^

### Blood sampling

Blood samples were drawn from the antecubital vein by means of careful venipuncture, using a 21 G sterile syringe without stasis. This was done between 08.00 and 10.00 AM after a fasting period of 12 hours. Glucose, creatinine and lipid profiles were determined using standard methods. Hemogram parameters were measured in blood samples collected in dipotassium EDTA tubes (Vacuette). An automatic blood counter (Beckman-Coulter Co, Miami, FL, USA) was used for whole blood counts.

### Statistical analysis

The Statistical Package for the Social Sciences software, version 16.0, was used in the statistical analyses of this study.

Categorical variables were expressed as frequencies (%) and were compared using the χ^2^ test. A Kolmogorov-Smirnov test was used to test the distribution of numerical variables. Those with normal distribution were expressed as the mean ± standard deviation and were compared using Student’s t test. Data without normal distribution were expressed as the median with the inter-quartile range (IQR) from the 25^th^ to the 75^th^ percentile, and were compared using the Mann-Whitney U test. In all statistical analyses, P-values < 0.05 were considered to be statistically significant.

Correlations between CHA_2_DS_2_-VASc risk score, presence of AF and other clinical, laboratory and echocardiographic parameters were performed using Pearson and Spearman correlation analyses, when appropriate. Univariate analysis on binary logistic regression was performed to identify which factors were associated with incident AF. After including each of these potential confounding factors, backward conditional binary logistic regression analysis was performed to estimate the odds ratio (OR) and 95% confidence interval (95% CI) for incident AF.

We carried out multivariate analysis on two models. Firstly, risk factors involved in the CHA_2_DS_2_-VASc score were excluded from this analysis to avoid multicollinearity. Secondly, risk factors and other factors except the CHA_2_DS_2_-VASc score were subjected to multivariate analysis. Receiver operating characteristic (ROC) curve analysis was used to analyze the prognostic value of the CHA_2_DS_2_-VASc score for new-onset AF, following STEMI. The C-statistic (area under the curve) was presented as a unified estimate of sensitivity and specificity. The area under the curve for AF was computed to identify the Youden index (best cutoff).^7^ The Youden index was deﬁned for all points of a ROC curve, and the maximum value of the index was used as a criterion for selecting the optimum cutoff point for detecting new-onset AF. According to the cutoff value that was obtained through ROC curve analysis, the study population was divided into two groups, named the low-risk and high-risk groups.

## RESULTS

A total of 696 patients (mean age: 62 ± 12 years; range: 23-92 years) with STEMI were included in this study. During the follow-up period, 70 patients (10.1%) developed AF. The demographic and clinical characteristics of the patients with and without AF are listed in Table 1. The patients with AF were older, and more of them were female, compared with the patients without AF (P < 0.001 and P = 0.011, respectively). While hypertension was more common (P = 0.002), smoking was less common among the patients with AF than among those without AF (P = 0.007). The diabetes mellitus, obesity and hyperlipidemia rates were similar between the patients with and without AF (for all parameters P > 0.05).

**Table 1.** Demographic and clinical characteristics of the patients with and without AF

	Without AF (n = 626)	With AF (n = 70)	P-value
Age (years)	61.8 ± 13	69.4 ±11	< 0.001
Body mass index	26.7 ± 4.3	27.5 ± 5.1	0.154
Heart rate at admission	77.4 ± 16.3	79.8 ± 18.3	0.256
Female gender (n, %)	117 (18.7)	22 (31.4)	0.011
Diabetes mellitus (n, %)	155 (24.8)	20 (28.6)	0.28
Hypertension (n, %)	275 (43.9)	44 (62.9)	0.002
Hyperlipidemia (n, %)	136 (21.7)	15 (21.4)	0.547
Smoking (n, %)	361 (57.7)	29 (41.4)	0.007
Ejection fraction (%)	45 ± 9.6	40 ± 9.8	< 0.001
Left atrial diameter (mm)	38 ± 4.1	39.7 ± 4.9	0.002
Location of MI			0.207
Anterior (n, %)	306 (49.4)	29 (43.3)	
Non-anterior (n, %)	314 (50.6)	38 (56.7)	
History of stroke (n, %)	7 (1.1)	3 (0.4)	0.07
Pre-hospital treatment			
Statins (n, %)	75 (12)	7 (10)	0.400
Beta-blockers (n, %)	104 (16.6)	14 (20)	0.285
RAS blockers (n, %)	108 (17.3)	5 (7.1)	0.016
Acetyl salicylic acid (n, %)	144 (23)	18 (25)	0.353
Clopidogrel	24 (3.8)	4 (0.6)	0.308
Hospital treatment			
Statins (n, %)	603 (96.3)	69 (98.6)	0.284
Beta-blockers (n, %)	590 (94.2)	61 (87.1)	0.02
RAS blockers (n, %)	554 (88.5)	52 (74.3)	0.002
Acetyl salicylic acid (n, %)	619 (98.9)	70 (100)	0.475
Clopidogrel (n, %)	576 (92.0)	62 (88.6)	0.217
Ticagrelor (n, %)	49 (7.8)	7 (10)	0.328
Amiodarone (n, %)	3 (0.5)	21 (30)	< 0.001
Total cholesterol (mmol/l)	173.2 ± 41.3	168.9 ± 39.4	0.41
HDL cholesterol (mmol/l)	40.3 ± 9	41.4 ± 8.4	0.46
LDL cholesterol (mmol/l)	107.2 ± 32.5	105.9 ± 29.3	0.71
Triglycerides (mmol/l)	128.8 ± 88.5	107.7 ± 32.5	0.002
BUN (mmol/l)	19.5 ± 6.8	21.5 ± 5.7	0.008
Creatinine (lmol/l)	1.0 ± 0.2	1.1 ± 0.2	0.56
CK-MB at peak (median)	170.9 ± 120	232.6 ± 209	0.002
Troponin T at peak (lg/l) (median)	4.6 ± 2.6	5.5 ± 5.1	< 0.001
Duration of hospitalization in the coronary intensive care unit (days)	2 ± 0.5	2.5 ± 1.3	< 0.001
Glucose (mg/dl)	169.6 ± 78.8	182.1 ± 82.3	0.212
CHA_2_DS_2_-VASc score	1.5 ± 1.4	2.7 ± 1.3	< 0.001
CHADS score	1.0 ± 0.9	1.5 ± 1.0	< 0.001

Data are presented as mean ± standard deviation or number (%) of the patients.

AF = atrial fibrillation; MI = myocardial infarction; RAS = renin-angiotensin system; HDL = high-density lipoprotein; BUN = blood urea nitrogen; CK-MB = creatinine kinase-myocardial binding; CHA_2_DS_2_-VASc = congestive heart failure, hypertension, age ≥ 75 years, diabetes mellitus, previous stroke, vascular disease, age 65 to 74 years, female gender.

The triglyceride levels were lower among the patients with AF than among those without AF (128.8 ± 88.5 versus 107.7 ± 32.5; P = 0.002), but there were no statistically significant differences between the patients with and without AF regarding other cholesterol parameters (for all parameters P > 0.05). The left ventricle ejection fraction was lower (P < 0.001) and the left atrial diameter was higher in the patients with AF than in the patients without AF (P = 0.002).

There were no statistically significant differences between the patients with and without AF regarding previous use of renin-angiotensin system (RAS) blockers, beta-blockers, acetyl salicylic acid, clopidogrel or statins. Use of in-hospital treatments, beta-blockers and renin-angiotensin system blockers was lower among patients with AF (P = 0.02 and P = 0.002, respectively), but use of other medications was similar between the patients with and without AF (P > 0.05). The patients with AF had a longer period of CICU follow-up than did the patients without AF (2.5 ± 1.3 versus 2.0 ± 0.5; P < 0.001).

The mean CHA_2_DS_2_-VASc and CHADS_2_ scores were significantly higher in the group with AF than in the group without AF (2.7 ± 1.3 versus 1.5 ± 1.4; P < 0.001; and 1.5 ± 1.0 versus 1.0 ± 0.9; P < 0.001, respectively).

### Binary logistic regression regarding incident AF

Univariate analysis showed that high CHA_2_DS_2_-VASc score, enlarged left atrium, high peak creatine kinase-myocardial binding (CK-MB) level, low left ventricle ejection fraction, long duration of hospitalization in the CICU, advanced age, female gender and history of hypertension were significantly associated with higher risk of incident AF (Table 2). On the other hand, use of renin-angiotensin system (RAS) blockers and beta-blockers in hospital was inversely associated with the risk of incident AF (Table 2).

**Table 2.** Univariate regression analysis of study variables

	Odds ratio	Confidence interval	P-value
CHA_2_DS_2_-VASc score	1.5	1.33-1.82	< 0.001
HT	2.1	1.29-3.59	< 0.001
Non-use of ACE blocker in hospital	2.6	1.47-4.80	0.001
Left atrial diameter	1.0	1.003-1.16	0.002
Peak CK-MB level	1.0	1.001-1.003	0.005
Duration of hospitalization in the coronary care unit	1.99	1.54-2.64	< 0.001
Female gender	1.99	1.15-3.43	0.013
Age	1.02	1.0-1.073	< 0.001
Left ventricle ejection fraction	0.952	0.92-0.97	< 0.001

CHA_2_DS_2_-VASc = congestive heart failure, hypertension, age ≥ 75 years, diabetes mellitus, previous stroke, vascular disease, age 65 to 74 years, female gender.

HT = hypertension; ACE = angiotensin converting enzyme; CK-MB = creatinine kinase-myocardial binding.

Multivariate binary logistic regression analysis was firstly conducted including the characteristics associated with new-onset AF in univariate analysis except for CHA_2_DS_2_-VASc score. This showed that no use of RAS blockers in hospital (OR: 2.40; 95% CI: ­1.25-4.53; P = 0.006), age (OR: 1.03; 95% CI: 1.017-1.062; P = 0.001), left ventricle ejection fraction (OR: 0.972; 95% CI: 0.94-0.99; P = 0.039) and duration of hospitalization in the CICU (OR: 1.63; 95% CI: ­1.19-2.23; P = 0.002) remained independent factors related to incident AF (Table 3).

**Table 3:** Multivariate regression analysis on study variables

	Model 1			Model 2		
	Odds ratio	Confidence interval	P-value	Odds ratio	Confidence interval	P- value
Non-use of ACE in hospital	2.40	1.28-4.53	0.006	2.16	1.14-4.10	0.017
Age	1.03	1.017-1.062	0.001			
Duration of hospitalization in the coronary care unit	1.63	1.196-2.230	0.0039	1.69	1.24-2.30	0.001
Left ventricle ejection fraction	0.972	0.94-0.99	0.002			
Peak CK-MB level				1.002	1.00-1.003	0.0024
CHA_2_DS_2_-VASc score				1.48	1.25-1.75	< 0.001

Model 1: risk factors and other factors except CHA_2_DS_2_-VASc score; Model 2: Variables except risk factors involved in CHA_2_DS_2_-VASc score.

CHA_2_DS_2_-VASc = congestive heart failure, hypertension, age ≥ 75 years, diabetes mellitus, previous stroke, vascular disease, age 65 to 74 years, female gender; ACE = angiotensin-converting enzyme; CK-MB = creatinine kinase-myocardial binding.

Following this, multivariate binary logistic regression analysis was then conducted including the characteristics associated with new-onset AF in univariate analysis except for hypertension, age and left ventricle ejection fraction. This showed that no use of RAS blockers in hospital (OR: 2.16; 95% CI: 1.14-4.10; P = 0.017), duration of hospitalization in the CICU (OR: 1.69; 95% CI: 1.24-2.30; P = 0.001), peak CK-MB level (OR: 1.002; 95% CI: 1.00-1.003; P = 0.024) and CHA_2_DS_2_-VASc score (OR: 1.48; 95% CI: 1.25-1.75; P < 0.001) were signiﬁcant predictors for new-onset AF. Furthermore, individuals with high CHA_2_DS_2_-VASc scores exhibited higher risk of incident AF than did those with low scores (Table 3).

### Prediction of incident AF

**Figure 2.**
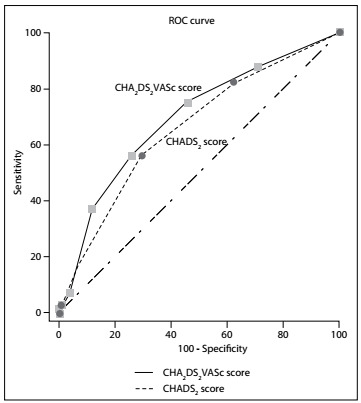
Receiver operating characteristic (ROC) curve with calculated area under the curve and optimal cutoff point for the CHA_2_DS_2_-VASc score and CHADS_2_ score, for identifying the presence of AF. C-statistic (area under the curve) and 95% conﬁdence interval (95% CI) for CHADS_2_: 0.663 (0.595-0.731); P < 0.001; and for CHA_2_DS_2_-VASc: 0.698 (0.631-0.765); P < 0.001. We calculated a cutoff point of 1.5 with the Youden index for CHADS_2_ and CHA_2_DS_2_-VASc scores, to estimate the presence of atrial fibrillation, with sensitivities of 56% and 75% and specificities of 71% and 54%, respectively.

ROC curve analysis showed that both the CHADS_2_ score (C-statistic: 0.663; 95% CI: 0.595-0.758; P < 0.001) and the CHA_2_DS_2_-VASc score (C-statistic: 0.698; 95% CI: 0.631-0.765; P < 0.001) were significant predictors of AF following STEMI (Figure 2). We calculated cutoff points of 1.5 for the CHADS_2_ and CHA_2_DS_2_-VASc scores, to estimate the presence of AF, with sensitivities of 56% and 75% and specificities of 71% and 54%, respectively.

According to the cutoff point of 1.5 that was obtained through ROC analysis, the patients were divided into two groups, with high and low risk. Both for higher CHADS_2_ score and for higher CHA_2_DS_2_-VASc score, the predicted risk of incident AF was higher: OR: 3.14; 95% CI: 1.89-5.22; P < 0.001; and OR: 3.72; 95% CI: ­2.10-6.57; P < 0.001, respectively.

According to the CHA_2_DS_2_-VASc score, the duration of hospitalization in the CICU was longer among the patients with higher risk than among the patients with lower risk (2.18 days ± 0.7 versus 2.04 days ± 0.4; P < 0.001). The time when AF started was earlier in the low-risk group than in the high-risk group (median of 5 hours versus 20 hours; P = 0.02). The time at which AF started and the duration of hospitalization in the CICU presented a correlation with each other (r: 0.698; P < 0.001).

## DISCUSSION

The main findings of this study indicated that CHA_2_DS_2_-VASc and CHADS_2_ scores were independently associated with the development of AF in patients presenting with STEMI. Consequently, both of these scores may be helpful and appropriate scoring systems for predicting AF following STEMI.

### Atrial fibrillation following acute coronary syndromes

Atrial fibrillation is the most common type of supraventricular arrhythmia following STEMI.^3^ Although AF that is developed following acute coronary syndrome is rare, it is associated with worse clinical signs and prognosis. Rapid management of arrhythmia is required in order to reduce the risk of complications.^8^

Left and right ventricular dysfunction, atrial ischemia, pericarditis, drugs, acute hypoxia and hypokalemia have been correlated with development of AF in the course of STEMI.^7^

In the GUSTO I trial,^9^ which included patients with acute coronary syndrome (AMI) who were eligible for thrombolysis, an incidence of AF of 10.4% was reported. Similarly, in the present study, the incidence of AF was 10.1%.

Among acute coronary syndromes that were logged in the Global Registry of Acute Coronary Events (GRACE),^10^ development of new-onset AF was predicted by older age, female gender, history of hypertension, presence of STEMI or non-STEMI, higher Killip class, higher heart rate, lower blood pressure, cardiac arrest on presentation and high initial serum creatinine levels.

In the Platelet Glycoprotein IIb/IIIa in Unstable Angina: Receptor Suppression Using Integrilin Therapy (PURSUIT) trial,^11^ AF was found more often in elderly patients with comorbidities like heart failure, hypertension and diabetes, and in those who were taking treatment with aspirin, oral anticoagulants, digoxin or antiarrhythmics before hospitalization. Patients with AF had higher heart rate at presentation, higher rates of ST depression, higher CK-MB levels and pulmonary edema. Similarly, in two studies, the predictors of AF in following up STEMI were found to be old age, female sex, higher Killip class, chronic kidney disease, large left atrium and low left ventricular ejection fraction.^11^ In our study, older age, large left atrium, female gender, low left ventricular ejection fraction, history of hypertension, higher peak CK-MB levels and long hospitalization in the CICU were determined to be predictors of AF.

According to the CHA_2_DS_2_-VASc score, the time taken for AF to start was longer among patients who presented high risk than among those presenting low risk. However, also according to this score, the patients who presented high risk spent longer times in the CICU than did the low-risk patients. Additionally, the length of time spent in the CICU and the time taken for AF to start showed a correlation with each other. Multiple risk factors may have contributed towards long times spent in the CICU. It is possible that new-onset AF continued to be diagnosed for as long as the stay in the CICU lasted.

Although it has been reported that pre-hospital treatment with RAS blockers and beta-blockers protects against AF following acute coronary syndrome,^13^ this was not the case in our study. Nonetheless, we found that in-hospital treatment with RAS blockers and beta-blockers had a protective effect against AF.

### CHA_2_DS_2_-VASc and CHADS_2_ scores and atrial fibrillation

The most relevant ﬁnding of our study was that CHADS_2_ and CHA_2_DS_2_-VASc scores are relatively strongly predictive of new-onset AF following STEMI. Previous studies had shown that both of these scores were associated with the risk of incident or recurrent AF. Yin et al.^14^ reported that CHADS_2_ and CHA_2_DS_2_-VASc scores were directly associated with the incidence of postoperative AF following valve surgery, and that higher scores were strongly predictive of postoperative AF. Barkas et al.^15^ reported that both scores were predictive of new AF in dyslipidemic patients. These findings were not surprising, since ­CHA_2_DS_2_-VASc and CHADS_2_ scores contain causal risk factors for AF.

Diabetes mellitus, hypertension, older age, congestive heart failure and cerebrovascular disease, which are components of CHA_2_DS_2_-VASc and CHADS_2_ scores, are associated with higher inflammatory status among patients.^16^ An association between inflammation and AF has been indicated in the literature.^17^^,^^18^ We previously reported that oxidative stress and inflammation parameters were associated with development of AF in patients presenting with STEMI.^19^ Inflammation may have provided a strong relationship between development of AF and both scores.

Increased left atrium size is the best-known predictive factor for AF.^20^ Studies evaluating patients with myocardial infarction have reported that these patients present greater incidence of AF, in relation to left atrial enlargement.^21^^,^^22^ The present study found a similar association between left atrial diameter and development of AF. Additionally, left atrial diameter was found to be an independent predictor for the development of AF. Hypertension causes structural changes, including left ventricular hypertrophy, impaired diastolic dysfunction and increased left atrial pressure and volume.^23^ Similarly, in the present study, hypertension was more often seen in patients with AF. Furthermore, left atrial diameter was greater in patients with AF than in patients without AF.

Although it has been suggested that better management of myocardial infarction will lead to improved outcomes for patients with AF,^24^^,^^25^ development of AF during STEMI still significantly influences short and long‐term mortality rates, including occurrences of sudden cardiac death.^26^ In the light of this evidence, our study may give rise to suggestions regarding screening for AF, especially among high-risk groups. Physicians should carefully screen for AF among patients with high CHA_2_DS_2_-VASc and CHADS_2_ scores and/or long hospitalization in the CICU. They should also screen for high peak CK-MB levels, especially among patients with symptoms of cardiac arrhythmia or with a diagnosis of thromboembolic cardiovascular events. Prompt management of arrhythmia is required, to reduce the risk of complications.

There are several limitations to our study. First, this study was observational and was conducted in a single center. Therefore, further studies are needed in order to reach definite conclusions. Lastly, our analysis involved a simple baseline determination at a single time point, but this may not reflect the patients’ status over long periods.

## CONCLUSION

CHADS_2_ and CHA_2_DS_2_-VASc scores predicted new-onset AF following STEMI. These data may inform AF screening strategies.
